# Organs-on-Chips Platforms Are Everywhere: A Zoom on Biomedical Investigation

**DOI:** 10.3390/bioengineering9110646

**Published:** 2022-11-03

**Authors:** Mohamed Zommiti, Nathalie Connil, Ali Tahrioui, Anne Groboillot, Corinne Barbey, Yoan Konto-Ghiorghi, Olivier Lesouhaitier, Sylvie Chevalier, Marc G. J. Feuilloley

**Affiliations:** Research Unit Bacterial Communication and Anti-infectious Strategies (CBSA, UR4312), University of Rouen Normandie, 27000 Evreux, France

**Keywords:** microfluidic platforms, organs-on-chips, tissue bioengineering, cellular microenvironment, disease modeling, personalized medicine

## Abstract

Over the decades, conventional in vitro culture systems and animal models have been used to study physiology, nutrient or drug metabolisms including mechanical and physiopathological aspects. However, there is an urgent need for Integrated Testing Strategies (ITS) and more sophisticated platforms and devices to approach the real complexity of human physiology and provide reliable extrapolations for clinical investigations and personalized medicine. Organ-on-a-chip (OOC), also known as a microphysiological system, is a state-of-the-art microfluidic cell culture technology that sums up cells or tissue-to-tissue interfaces, fluid flows, mechanical cues, and organ-level physiology, and it has been developed to fill the gap between in vitro experimental models and human pathophysiology. The wide range of OOC platforms involves the miniaturization of cell culture systems and enables a variety of novel experimental techniques. These range from modeling the independent effects of biophysical forces on cells to screening novel drugs in multi-organ microphysiological systems, all within microscale devices. As in living biosystems, the development of vascular structure is the salient feature common to almost all organ-on-a-chip platforms. Herein, we provide a snapshot of this fast-evolving sophisticated technology. We will review cutting-edge developments and advances in the OOC realm, discussing current applications in the biomedical field with a detailed description of how this technology has enabled the reconstruction of complex multi-scale and multifunctional matrices and platforms (at the cellular and tissular levels) leading to an acute understanding of the physiopathological features of human ailments and infections in vitro.

## 1. Introduction

Around the globe, scientists are working on developing new solutions and exploring new alternatives to beat the insufficiency of the current preclinical assessment protocols and to overwhelm critical issues directly associated with disease modeling, inquiries about in vivo drug toxicity and the complexity of personalized medicine. These solutions, designated as Integrated Testing Strategies (ITS), can combine in silico, in chemico and sophisticated in vitro approaches and have been particularly promoted by the Organization for Economic Cooperation and Development (OECD) in response to the UE REACH program [1]. It is believed that many in vitro dilemmas can be overcome with the advent of organ-on-a-chip (OOC) and human-on-a-chip (HOC) platforms. Amongst a long list of diverse biomedical investigations, OOC modes have gained significant research attention in recent years, with the more specific target of designing and engineering miniaturized functional devices of human tissues and organs [2,3,4,5]. OOC represents the marriage of tissue bioengineering and microfluidics technologies. The implementation of the latter in OOC helped in the development of a complex physical microenvironment mimicking the physiological systems in a microscale range. These micro-architectures promise to better emulate native biological functions ex vivo and allow high throughput approaches crucial to disease modeling, personalized medicine, and drug testing. Central to this sophisticated technique is the use of perfusable microfluidic platforms, in which fluid flow both sustains the metabolic needs of OOC biosystems and replicates the distinct vascular compartment found in living organisms [2].

Herein, we examine recent innovations in the field of organ-on-a-chip state-of-the-art technology coupled with other related disciplines, encompassing microfluidics, tissue bioengineering, technologies for replicating cell differentiation, and their structure and function at the in vivo scale. We will also discuss different OOC platforms and biosystems that have advanced our knowledge of fundamental characteristics of miniaturized vascular biology, particularly in terms of exploration of tissue-level functions in organ-specific models. We wrap up this review with recommendations about how to select the most adequate OOC system in terms of the appropriate cell line, envisaged tissue, and organ function, in order to accurately target vascular biology questions.

## 2. Microfluidics: A Piece of Literature

Concomitantly considered as a science and a technology, microfluidics allows for handling and processing reduced reagent volumes and vastly reduced instrumental footprints. It is commonly used to monitor, manipulate and process simple or complex monophasic or multiphasic fluids circulating through a network of microchannels, oscillating from tens to hundreds of microns in terms of size or confined within micro-chambers, that are commonly known as ‘lab-on-a-chip’ [6,7,8,9,10]. The microchannels are characterized by large surface areas and high mass transfers. They allow the use of low reagent amounts, controllable volumes, fast mixing speeds, rapid responses, controllable pressure levels, and precision control of physical and chemical properties, heralding a new era in biomedical investigations, personalized medicine and drug delivery strategies [11,12,13,14]. Microfluidics integrate various operations such as sample preparation, reactions, separation, detection and basic operating units such as cell culture, sorting and cell lysis [15].

From a physical viewpoint, such discipline helped to elucidate and understand several terms in biophysics and its diverse applications, encompassing laminar regime flow, rapid diffusion time, and even control of fluid behavior via miniaturized microtubing. [16,17]. Within every single device of lab-on-a-chip, it has been demonstrated that the detection can be attained at a nanoliter scale, reflecting higher control of media flows and higher precision of the experimental trials. Such miniaturized and sophisticated designs can ensure multiple concomitant treatments and the modeling of a wide range of complexities in terms of biological processes at tissue and organ levels [18,19]. Nowadays, the staggering advancement of OOC technologies has promoted the microfluidic discipline to be used in various fields, encompassing biotechnology, cell and tissue bioengineering, pharmacology, drug screening, drug delivery, biomedical sciences and mimicking human health on microfluidic chips [20,21].

The development of microfluidics in the scope of microscale devices experienced a burgeoning growth, leading to strong support of the emergence of ‘organ-on-a-chip’ technology [22]. The latter uses these microstates of fluids (10^−9^ to 10^−18^ L) to drive them through designed microchannels (10–100 μm), aiming to mimic the physiological path-ways in the human body [23,24]. For these reasons, interest in OOC has astonishingly intensified in an exponential way. During the decade of the 2000s, a constant number of developments allowed the scaling down of the macroscale arrays, metamorphosing them into miniaturized models, also known as ‘chips’ [22]. The technology circumscribing microfluidics was henceforth blended, not only with the various fields of smart microelectronics, advanced molecular analytics, computational biology, but also in the realm of ‘organs-on-chips’ [22,25]. The latter, combining a wide range of chemical, biological and material science disciplines, was selected as one of the ‘Top Ten Emerging Technologies’ in the World Economic Forum [26].

The microfluidic systems in on-chip platforms enable controlled microliter laminar fluid flow [12], separation, mixing, and rapid biochemical and genetic analyses with the highest degree of precision [27]. Such state-of-the-art technologies also allow for biomarker-identification, drug screening and its delivery [28] and even real-time imaging [29]. Another potential that may be attributed to the microfluidic discipline is that the latter may ensure the integration of diverse types of sensors into the microfluidic chip [30] leading to the real-time tracking of results. Such miniaturized devices make them portable and adaptable to perform various trials and arrays [3]. Moreover, the use of small volumes, at micro- and nanoliter scales, of fluids has greatly lowered the cost of micro-architecture microfluidic devices. The latter can also be used to manage the vulnerable field of stem cells, their microenvironment and differentiation [31,32] with potential use at a larger investigative scale with expanded accurate applications. According to Langer and Vacanti [33], tissue engineering (TE) is highly based on the natural biology of a system in order to ensure the development of strategies to replace, repair, maintain or even enhance the function of an existing tissue, using new viable cells or bioengineered tissues and organs. Nonetheless, it is pertinent to note that cell culture represents the salient prerequisite for tissue and organ culture. For such a reason, synergism between microfluidics and tissue engineering seems to be in favor of cell manipulation within microphysiological systems.

The most critical consideration for in vitro models is the reproduction of a desired in vivo organ via the selection of a suitable cell type from an appropriate cell source. Over the decades, tissue engineering has highly relied (and still does) on animals as the main source of cells; however, this deeply fails to simulate human functionality and complexity [28]. In the same line, Huh and his group [34] showed that the use of animal cells results in only limited success in representing human disease. It is relevant to know that the human cells utilized in TE, particularly in OOC platforms, belong to various categories of cells encompassing immortalized cell lines, primary cells, stem cells and their differentiated progeny.

Generally, classic approaches for tissue engineering have been limited to two-dimensional (2D) monolayer cell cultures, mostly in conventional Petri dishes, whose success is tightly related to various key parameters of the biological and physiologic characteristics of the environment. In 2014, Bhatia and Ingber [29] succeeded to demonstrate that the use of 2D tissue culture may lead to the imminent loss of the distinct phenotype, normal tissue micro-architecture, and tissue-related techno-functionalities may take place because of the huge gap between the in vitro and in vivo conditions. Based on the use of scaffolds and hydrogels, the next dimension of cell culture, also known as 3D cell culture, has surmounted several hurdles in this field, but other impediments emerged, especially in terms of lack of vascularization, low diffusion properties, insufficiency of oxygen, and nutrient supply with a significant decrease of appropriate cellular differentiation and growth [35,36,37,38].

## 3. Organs-on-Chips: The State-of-the-Art Technology

Nowadays, the most critical challenge is to better emulate human physiology, biological functions and human pathophysiology at multi-scales oscillating from the molecular to the cellular, tissue, organ or even whole organism level. Monolayer cell cultures and animal models represented (and still do) the main basis of the current model systems. The pros of simplistic monolayer cultures also known as 2D cultured cells are associated with the presence of several cons, notably in terms of the significant difference in gene expression, epigenetics, cell morphology alteration, cell flattening with cytoskeletal and changes in shape of nucleus [39,40], cell function compared to native 3D tissues, and lack of cell–cell and cell–matrix interactions, [41,42] which may lead to the absence of tissue-specific properties.

Within the same context, it has been revealed that the use of animal models in biomedical investigation failed to provide accurate human responses at a pathophysiological level, a critical issue mainly due to significant species divergences [43]. Conventional in vitro models are straightforward, robust, and suitable for high-throughput research, but their biological relevance to the complex human body tissues is limited. Such hurdles played the role of an impetus to urgently develop substitute models, also known as cell-based 3D models, at in vitro scale, that better resemble the intricate biological circuits and complex functionalities of living organs. Further, 3D cell cultures technologies, with their various designs, opened the gate to a list of improvements in cell research, reflecting a more accurate portrayal of cellular processes. Tridimensional cell cultures helped in subverting research on animal models, thus making the research more humane. Likewise, investigations in drug discovery and cancer performed on 3D cell cultures unveiled more accuracy; even the gene expression correlation seems to be better with such models [44]. Nevertheless, it is relevant to know that every new technology brings newer challenges and limitations. The main encountered hurdles with the 3D culture include the presence of several human-derived hormonal components within matrices and/or scaffolds, leading to less adaptability to a clinical set up, accompanied by difficulty in terms of 3D culture detachment [45], batch to batch variability, and nucleic acids/proteins isolation from 3D cell cultures [46].

The next step in the ladder of scientific progress and research was the appearance of the first-generation of microfluidic biological designs, also known as ‘cells-on-a-chip’, where such devices contributed to the understanding of micro analytics and molecular analysis (such as microarrays) [47]. In the same line, the coupling of microfluidics and 3D cell cultures allowed a tremendous step in the field of cell biology research, reflecting an imminent improvement of emulating the in vivo cell microenvironment. Such amalgamation adds multiple dimensions to cell research at diverse scales, encompassing miniaturization, adaptability towards a high throughput design, easiness of use, high sensitivity and robustness [48]. Here comes the technology of organ-on-a-chip. To address critical biomedical issues, such technology, developed from in vitro cell growth to circumvent in vivo system restrictions, seems to be the most relevant alternative that can reproduce particular organ biological functions [49,50].

Regarding the key components of the OOC technology, Mandenius [51] defined four pivotal sections of this cutting-edge technology, encompassing (*i*) microfluidics discipline; (*ii*) living cell tissues; (*iii*) stimulation or drug delivery and (*iiii*) sensing. This part of this technology is highly based on miniaturization, integration, and automation [15]. As regards the cell tissue section, it relies on specific components firmly related to the cell line, notably in bi- or tridimensional systems. Within this field, hydrogels seem to be the most used tool in such cell arrangements showing a high preventive potential of cell mechanical damage [52]. Within this context, Table 1 recaps two decades of the main stages of the organ-on-a-chip technology from its first step to its most advanced highlights.

The intensive study of tissues revealed that physical and/or chemical signals are, for certain typical tissues, requisite to emulating the physiological microenvironment, which leads to micro-tissue maturation and function such as the case of how electrical stimulation may contribute to the maturation of the myocardial tissue [53]. Likewise, it has been shown that different signal stimuli can be derived from drug screening approaches [54]. The sensing component is used to detect and acquire data and may be an embedded sensing component or a transparent chip-based visual function appraisal system. Within this framework, Peel and collaborators [55] succeeded to use automated systems in order to view multicellular organ-on-a-chip, reflecting highly exhaustive cell phenotypes and statistical standards for different dimensions. In the same year, Kane et al. devised a cell system to examine diverse cell lines in a 3D microfluidic arrangement [56]. To wrap up, a significant human-on-a-chip cell model cannot be described and accessed without the presence of microsensors-mediated measurement of metabolic state at specific spots in the device.

## 4. Organs-on-a-Chip: A Paradigm Shift in Biomedical Investigation

The perfect couple of microfluidics and OOC technology have developed swiftly in recent years, enhancing our knowledge of almost all the major organs, even underestimated organs such as the skin [57,58,59], pancreas [60], skeletal muscles [61,62], blood vessels [63,64,65], brain and blood–brain barrier (BBB) [66,67,68] and central nervous system (CNS) [69,70].

Herein, we sum up some examples of various organs with the associated chips, their main hallmarks, their performances and their major utilities (Table 2).

### 4.1. Heart

Cardiovascular deaths represent a heavy burden to public health, occupying the top listed cause of human mortality. Microfluidics technology and its nonstop surfacing has enabled in vitro bionic investigations of cardiac tissue, where the myocardium is a major component of the heart. According to Visone et al. [84], the beating of cardiomyocytes (CMs) can be used in microfluidics to directly assess drug effects and is directly related to heart pumping. Grosberg and coworkers [85] used polydimethylsiloxan (PDMS) in order to produce an elastic film with a surface texture, leading to an implantation of neonatal rat CMs on the membrane forming muscle membranes. The contraction of cardiomyocytes leads to the curl of the muscle film to one side. The analysis of the differences in terms of size of the cell contractile potentialities was highly feasible via the measurement of the curl’s degree on the PDMS film. It is relevant to know that the experimental device was appropriate for both single-muscle membrane measurements and high-throughput automated multi-plate assays. Likewise, Zhang et al. [86] succeeded to produce self-assembled myocardial sheets in a PDMS model using hydrogels. The myocardial cells were derived from differentiated myocardium. In the same framework, 3D-printing technology opened the gate to researchers to produce micro-organ tissue chips, leading to the integration of myocardial and vascular systems [87]. The system utilized vascular endothelial cells to form vascular intricate complex schemes. Then, cardiomyocytes were added to the vascular network gap. The organ-on-a-chip produced a screening platform for cardiovascular-related drugs. In 2016, Zhang et al. [88] unveiled the heart-on-a-chip device using high-speed impedance detection in order to assess cardiac drug delivery and efficiency. This state-of-the-art system recorded the contraction of cardiomyocytes to accurately reveal different drug pathways and their effects. The produced chip represented a preclinical appraisal of drug cardiac efficacy.

In the same line, a heart-organ platform has been developed by Marsano et al. [89], emulating the physiological mechanical environment of cardiomyocytes. This complex vascular network has been directly visualized and quantitatively analyzed, an option that was not allowed in traditional cell culture or in animal models. Such a platform corresponds to a highly sophisticated technology, reflecting a real advancement in this realm providing standard functional 3D heart models.

This scientific progress in terms of disease modeling and personalized medicine makes the mechanism an innovative and low-cost screening platform, improving the predictive potential of in vitro models. In 2019, Schneider and collaborators [90] worked on creating efficient chips generating heart tissue in a controlled microenvironment based on human-induced pluripotent stem cells (hiPSC). The rhythmic function and viability of myocardial tissue were maintained for an extended time period, in addition to a specific optical detection of detailed spatiotemporal pulsation dynamics. Such a platform represents a tremendous step in the world of personalized medicine and biomedical investigations. In 2016, Tzatzalos et al. [91] reported that the hiPSC cardiomyocytes can represent an outstanding potential for healthy and disease-specific cardiomyocytes to appraise the efficiency of drugs for dilated cardiomyopathy. Such progress in the field of drug delivery and development reflects crucial implications for cardiovascular tissue because cardiotoxicity is often seen in drug trials and is one of the main reasons leading to imminent clinical trial suspension or urgent withdrawal of drugs from the market.

### 4.2. Brain

The brain is one of the most sophisticated and intricate organs, particularly in terms of the variety and specificity of cells that it contains. It is relevant to know that human brain genetics and functions are drastically different from other animals. Hence, animal models can only provide us with a shallow understanding of brain functions and disorders [92]. Microphysiological neural systems-on-chips (NSCs), also commonly termed brains-on-chips (BOCs) or spinal cords-on-chips (SOCs), correspond to an emerging critical area within the research field of microfluidics and OOC technology because of the disproportionate adverse effects and the heavy burden of neurological ailments and disorders on society. These microfluidic BOCs platforms have been established to better emulate in vivo conditions, including chemical, electrical and physical conditions of the human brain [29,93]. According to Menken et al. [94], neurological diseases and disorders account for 8 of the 10 most disabling ailments, as defined by the World Health Organization (WHO). In the last decade, the outstanding advancement in microphysiological systems and microfluidic technology facilitated analyses of these diseases and disorders with heavy public health impact, which are difficult to achieve with traditional cell culture methods or animal models [18]. Moreover, Mak et al. [95] demonstrated that OOCs can match well with human cells, supplying a potential preclinical screening alternative to animal models.

Owing to the importance of axons in neurodegenerative ailments, some scientific researchers and clinicians only focused on neuronal axons. In this field, Taylor et al. [96] developed a microfluidic micro-architecture for high-resolution axonal transport. This platform ensured the isolation and monitoring of axonal mitochondria and axonal growth. Likewise, a circular microfluidic device designed by Park et al. [97], in which the soma section was located in the center with sealed microgrooves separated from the axonal section. From these microgrooves, a straight pathway for axonal growth emerged. This research group has established a specific imaging technique to quantify axonal growth, solving the problem of invasive sampling to characterize axonal growth. Diverse EMC components counting collagen, laminin and matrigel revealed various effects on the growth of axons and soma compartments, separately. Firmly associated to different parts of the neurons, their exposition to these biomolecules unveiled distinct effects on axon growth.

In 2011, Kunze et al. [98] constructed a microfluidic device for neural cell culture to construct neural layers and 3D architecture. They described agarose–alginate mixtures that build multilayered scaffolds with layers of embedded primary cortical neurons apart from cell-free layers. The delivery of B27 ensured the formation of concentration gradients. Consequently, this 3D scaffold-based microdevice represented the basis for in vitro trials and drug testing. In harmony with this field, Park et al. [99] produced a multilayer microfluidic system to emulate in vivo brain microenvironment for neurodegenerative diseases and high-throughput drug testing. This team succeeded in cultivating pluripotent human cells on a chip to incorporate the blood–brain barrier (BBB). The complex cellular interactions between human fetal neural progenitor cells and the mature model have been assessed in this micro-architecture. The use of an osmotic micropump allowed us to investigate and assess the effect of flow on neurodevelopment.

In 2016, a state-of-the-art silicone elastomer brain-on-a-chip in vitro model was designed by Kilic and coworkers [73], where neurospheroids were cultured on this miniaturized microdevice with flow control. It is relevant to notice that changing the flow within the platform led to an obvious intricate neural network and neural differentiation. Various parameters have been tested and assessed, encompassing toxic effects of amyloid-β in two different conditions (with and without flow). The findings showed that neurospheroids develop better in the dynamic conditions. Likewise, Dauth et al. [100] succeeded in the fabrication of a multiregional brain-on-a-chip platform that permitted the development of particular disease models. Amongst the outcomes, a significant reduction of firing activity and change in the amounts of astrocytes and particular neuronal cell types compared to separately cultured neurons was obviously noticed. The direct impact of phencyclidine (known as angel dust), which is a mind-altering drug, has been investigated in this microfluidic system as well.

As a critical field of medical investigation, notably in terms of neurological diseases and disorders, the blood–brain barrier (BBB) has been modeled widely with microfluidic brain-on-chips advancement. Such targeted chips are micro-architecture devices, which ensure modelling of the flow conditions within the blood–brain barrier [68,101,102,103]. For instance, Booth and Kim [101] designed a brain-on-a-chip model for BBB investigation. They use a PDMS two-channel microfluidic device with a semi-permeable membrane coated with bEnd.3 brain endothelial cells on one side and C8-D1A astrocytes on the other. The same team performed transendothelial electrical resistance (TEER) experimental trials by placing miniaturized specific electrodes on both sides of the membrane. The experiment indicated the development of tight junctions within a stable BBB model. Always with this miniaturized system, these two researchers [86] have measured cell permeability by flowing diverse molecular-weight fluorescein isothiocyanate (FITC)-dextran through the endothelial cell microfluidic channel and measuring the FITC-dextran concentration on the astrocyte side of the membrane. The promising findings revealed high similarity with previous BBB models in terms of permeability, reflecting a high possibility of using such a system in drug-delivery investigations. Likewise, Griep et al. [102] devised another BBB platform. They used an analogous microfluidic design containing a transwell membrane coated with hCMEC/D3 human BBB endothelial cells. This device functionalized with TEER electrodes in purpose to examine the impact of shear stress and chemical stress on BBB function. It is crucial to note that the addition of tumor necrosis factor (TNF) α result-ed in reduced barrier tightness [102].

At the same year, Prabhakarpandian and collaborators [103] designed another microfluidic BBB platform, based on the use of microgaps to separate the two microtubules: one containing a brain endothelial cell line (RBE4) and the other containing astrocyte-conditioned media. According to this team, FITC dextran permeation studies and the presence of tight junction proteins suggested a stable and functional BBB model. The creation of three-dimensional hydrogel systems containing integrated microfluidic channels, offering a new promising path for constructing biomimetic brain-on-chips, neural systems-on-chips and spinal cords-on-chips. Recent massive efforts using microfluidic-based blood–brain barrier models have evolved from incorporating extracellular matrixes (ECMs), replicating hydrogels instead of transwell membranes or filters [68,104].

In the monarchy of cancer therapy, magnetic hyperthermia therapy attracted researchers’ attention in cancer therapy due to the next generation of local heat to highly reduce damage to nearby healthy cells and optimize the treatment. In 2020, a highly sophisticated miniaturized microfluidic-based brain tumor-on-a-chip platform has been designed by Mamani and collaborators [105] with the purpose of accessing the therapeutic effect of magnetic hyperthermia on the chip. This microdevice allowed scientists to cultivate 3D glioblastoma cells in the central zone of the microfluidic channel. Cell viability after exposure to magnetic hyperthermia therapy has been investigated on the microsystem. Results of fluorescence imaging have shown astonishing findings in terms of a 100% decrease of cell viability after 30 min of exposure to magnetic hyperthermia therapy. Knowing that tumor vasculature is absent in this micro-architectural model, the results of this investigation revealed that this brain tumor-organ-on-a-chip platform has great potential for imitating the main features of glioblastoma brain tumors in vitro.

### 4.3. Liver

According to Mccuskey [106], the hepatic system represents the salient site of drug/toxin metabolism in the whole human body. This noble organ, the liver, constitutes a series of complex hepatic lobules that confer multicellular functional communication. The challenge within the hepatocytes culture is the maintenance of their physiology and integrity over an extended time period [107]. Within this field, in 2006, Kane and colleagues [108] were the first to design a liver-based system with microfluidic pores in which 3T3-J2 fibroblasts and rat liver cells were co-cultured to emulate an airway interface. Amongst the astonishing findings, rat hepatocytes cultured in the chip revealed a continuous and stable synthesis of albumin with a normal metabolism. A year after, Lee et al. [109] succeeded in designing a chip that reflected the interstitial structure of endothelial cells, in which they were able to culture primary hepatocytes using perfused culture media outside the permeable endothelial gap. The latter separated hepatocytes in cord-based structures, permitting their separation from the external sinusoidal compartment and concurrently maintaining active substance exchange. In 2013, Ho and coworkers [110] led a pilot study in which they used radial electric field gradients produced using electrophoresis to pattern cells onto circular PDMS chips. These novel approaches highly mimicked the hepatic lobule structure. In the same line, Hegde et al. [111] bioengineered a two-layer chip using a porous membrane of polyethylene terephthalate (PET). This microfluidic device has been constantly perfused by collagen and fibronectin-sanded rat primary hepatocytes into the lower channel via the upper compartment.

To ameliorate the physiological models in terms of efficiency and adaptability, specific (3D) tridimensional hepatocyte culture tools were used from microfluidic chip technology [112]. Yum et al. [113] produced systems to investigate accurately how hepatocytes affect other cell types. High-throughput assays were developed to assess liver cell drug toxicity. In 2016, Riahi and his team members [114] produced microfluidic electrochemical chips with immuno-sensors to ensure the highest detection of the biomarkers produced during hepatotoxicity. Two years later, Ma and collaborators [115] produced a biomimetic platform for the perfusion of hepatic spheroids in situ. In the same year, Chong et al. [116] developed diverse assays to scrutinize drug skin sensitization via the appraisal of metabolite production and the activation of antigen-presenting cells (APCs). This technology holds value as a drug screening platform in order to identify components responsible for systemic skin reactions. An astonishing study was performed by Lu et al. [117] in which diverse biomimetic liver tumors were developed via integrating decellularized liver matrixes (DLM) with gelatin methacryloyl (GelMA) to recreate the 3D tumor microenvironment (TME). This system represents the basis of a whole package of future anti-cancer pharmacological investigations, reflecting the real mirror of modeling disease and personalized medicine. Moving forward with this technology, a wide range of liver diseases and injuries has been applied and tested. In this line, Kang and colleagues [118] used the liver-on-a-chip to analyze the viral replication of the hepatitis B virus. In the same year, Zhou and his team [119] succeeded in developing a system for modeling alcohol injury. It is pertinent to notice that further drastic characterization of cultured cytoplasm in terms of metabolomics, proteomics, genomics, and epigenomic analysis will highly improve the functional outcome of these investigations.

### 4.4. Lung

Every single OOC has its unique requirements, though applications associated with the respiratory tract always have tremendous impact. For instance, when the lungs are infected by fine particulate matter, bacteria, viruses and/or any other type of exogenous factors, white blood cells accumulate, and the mucus produced blocks the airway. These vital biophysiological steps and processes are so sensitive to observe in animals; thus, the need for developing lung-on-a-chip technology is urgently reported. As a worldwide pioneer in the realm of in vitro organ-on-a-chip development, the Wyss Institute at Harvard University was the first to develop the lung-on-a-chip, a device first of its kind in the world [18]. The chip was completely based on PDMS, a polymeric organo-silicon compound, with an upper and a lower layer of channels separated by a porous membrane and coated with extracellular matrix. This device is highly well structured internally. It contains an upper layer. The latter consists of alveolar epithelial cells, allowing gases to pass through, whereas the lower layer comprises microvascular epithelial cells that permit white blood cells to pass through, thus emulating lung function. This technology was used for the first time by Benam and coworkers [120] in order to test smoking and non-smoking conditions. This investigation confirmed that using the lung-on-a-chip yielded experimental findings that were closer to clinical physiological and inflammatory reactions, compared with those from animal experiments. It also led to the discovery and analysis of biomarkers that were even more accurate [120]. During this period, a nonstop wave of advancement in the field of lung-on-a-chip, in terms of various designs, different structures and specific physiological responses, has swiftly emerged [121,122,123,124,125]. The lung-on-a-chip, a state-of-the-art device has been designed and developed to show its relevance in drug delivery, development of medicines and disease modeling. Nevertheless, it still has quite a few practical obstacles that must be conquered if such devices are to be used in applied toxicology research [126,127]. The aim of surmounting these challenges is to continuously ameliorate the usability of these devices and to emulate metabolism in the human body more accurately.

### 4.5. Kidney

As a noble organ, the kidney represents one of the most sophisticated organs in the human body. Its micro-architecture is of pivotal importance in terms of filtration of blood, toxin removal and maintenance of electrolyte balance, and it is still hard to emulate since it is comprised of an intricate net of different tissues. Drug development has resulted in severe kidney toxicity as one of the most reported adverse effects. Jang et al. [79] were the pioneers who developed a kidney-on-a-chip platform. The latter contained a perfusion circuit in order to develop primary kidney proximal tubular epithelial cells under microfluidic conditions. The dynamic flow seems to be the key element of the human kidney proximal tubule. Accordingly, this microfluidic kidney-on-a-chip system was highly efficient in terms of ensuring alkaline phosphatase activity, reabsorption of glucose, and transport of albumin. In the same context, Wilmer et al. [128] published an excellent re-view with focus on the recent advances of kidney-on-a-chip systems particularly how they mimic the structural and techno-functional properties of the human kidney in purpose to predict some specific trials such as drug-induced kidney injury.

The study of glomerulus, as the salient micro-architecture in blood filtration, has encouraged researchers to design a correlating model. In 2017, Musah and his group [32] succeeded in the design of a kidney-glomerulus-on-a-chip microdevice. This model contained human iPSCs that differentiated into podocytes. The latter correspond to the class of cells that regulate selective permeability in glomerulus. A glomerulus-on-a-chip platform has been devised in order to simulate adriamycin-induced albuminuria and podocyte injury. Similarly, in 2016, Zhou et al. [129] developed a sophisticated micro-architecture kidney-glomerulus-on-a-chip with astonishing potentialities, especially in terms of mimicking the epithelial and vascularized interface between podocytes and endothelial cells within the kidney glomerulus units. To reconstruct the glomerular microenvironment, particularly in terms of cellular cytoskeletal rearrangement and cellular damage in kidney disease, the injection of dynamic flow coupled with mechanical forces seems to be the crucial parameter to make the chip work, reflecting the importance of shear stress and hydrodynamic pressure in such a miniaturized device. In the same line of kidney ailments, Wang et al. [130] designed a virus-induced kidney disease system on the basis of a three-layer microfluidic chip. This distal tubule-on-a-chip micro-architecture model was conceived to examine the pathogenesis of virus-induced renal dysfunction in the regulation of electrolytes.

### 4.6. Skin

Loaded with an arsenal of vital functions, the skin, as the largest human organ, plays a key role in the regulation of body temperature, prevention of dehydration and protection of the human body against pathogens and exogenous stress factors. According to Jahanshahi et al. [131], proceeding toxicological assessment tests of new compounds, particularly in the dermatology discipline, require physiologically relevant skin models. The latter are so crucial for pharmaceutical, chemical and cosmetic industries to identify potential hazards on the skin. Herein, we shed light on the current advancements in skin-on-a-chip models. One of the key traits to develop biomimetic skin models is to reproduce the micro-architecture of vasculature and blood circulation in vitro. As a result, the combination of two state-of-the-art technologies, tissue bioengineering with microfluidic technology, by providing the option of media perfusion, is expected to recreate more relevant skin platforms and provide a valuable assessment of drug experiments [132]. In 2013, a pilot investigation was performed by Wagner and collaborators [133] in which this group succeeded in developing a microfluidic platform incorporated with a peristaltic micropump for co-cultures of human artificial liver microtissues and skin biopsies. They were able to highlight tremendous outcomes in terms of observation of a crosstalk in the co-culture during a long-term period of exposure to fluid flow (14 days). Within this system, tissue sensitivity has been investigated via exposure to a pharmaceutical substance, troglitazone. Single epidermal layer models are face-to-face with in vitro skin models with dermal and epidermal layers, the latter win the battle in terms of relevance and reproduction. In 2015, Abaci and his group [134] devised a skin-on-a-chip microfluidic device with exceptional potential in terms of the particular capability of recirculating the culture media without the need for pumps or external microtubing for controlled flow volumes. Within this sophisticated design, it is pertinent to note that physiological residence time of blood in the skin tissue has been accurately studied and recognized in order to provide the appropriate concentration of drugs in the blood stream. Such a miniaturized device is used for toxicity drug testing and assessment.

In the field of biosensors, the latter, once incorporated with biochips, provides in situ and real-time monitoring of skin tissue responses to the trial item. Similarly, Alexander and coworkers [57] designed a highly sophisticated skin-on-a-chip platform integrated with a biosensor for monitoring the transepithelial electrical resistance (TEER) of reconstructed human epidermis. It is relevant to notice that metabolic parameters and change of skin tissue micro-architecture over time have been regularly checked in this platform. Kwak et al. [135] successfully accomplished the fabrication of a skin-on-a-chip microfluidic platform in which epidermal and dermal layers were co-cultured with human umbilical vascular endothelial cells. The UV irradiation and the exposure to the doxorubicin of skin tissues have been followed up by an intensive immune response in terms of a secretion of cytokines and migration of neutrophils into the dermal layer.

In the same vein, a three-layer PDMS microfluidic skin-on-a-chip system with two porous membranes has been designed by Wufuer et al. [80]. In this platform, a co-culture of diverse human skin cells (epidermal, dermal and endothelial layers) has been developed. This system contains intricate separate microfluidic channels that supply the ability to inject a wide range of culture media with different flow rates. An epic experimental trial consisting of the perfusion of tumor necrosis factor alpha into the channels has led to an emulation of the skin inflammation and edema in this system. Additionally, this model permitted the investigation of dexamethasone’s effect on reducing inflammation and edema via a drug toxicity assessment approach. However, this dynamic and multilayer co-culture system lacks the 3D microenvironment of the skin.

In 2017, Lee et al. [136] succeeded in the design of a skin-on-a-chip model, which is mainly based on a tridimensional co-culture. In this system, dermal primary fibroblasts have been established in hydrogel structures in order to provide a 3D dermal layer. Subsequently, the step of primary keratinocytes cultures on top of the collagen-fibroblast layer was performed to reconstitute the epidermal layer. In this biochip, the role of a microfluidic microtubules coating was shown via good growth and differentiation of skin cells. Likewise, Jusoh et al. [137] developed a miniaturized design of a skin-on-a-chip model to study the impact of skin irritants on angiogenesis. Within this biochip, irritated keratinocytes biochemically mimic vascular endothelial growth factors. Such a result led to imminent angiogenic growth through the interactions between autocrine and paracrine and dermal fibroblasts and keratinocytes. It is important to know that the effect of the sodium lauryl sulphate, also known as SDS reagent (a well-known chemical irritant), and steartrimonium chloride (which is known as a non-irritant compound) has been investigated and tested in this microphysiological system. Such a field of research has attracted nonstop interest. In the same context, Jahanshahi et al. [131] designed a gelatin-based skin-on-a-chip device to study wound infection, the skin’s pro-inflammatory response and drug screening. Such a biochip ensured the culture of keratinocytes on microtubules, which have been fixed in a gelatin matrix. A period of six weeks of cell culturing was critical to form the multilayer structure of the epidermis layer. However, a drawback of this system has emerged, particularly in terms of the absence of other cell types (e.g., fibroblasts), even though this model is still a functional prototype for studying the skin’s pro-inflammatory responses to bacterial infection and drug testing.

To wrap up, the development of more reliable skin models and platforms to emulate the complexity of the 3D micro-architecture of human skin, the presence of vascular intricate network and immune cells is pivotal for toxicology assessment and studying skin disorders.

### 4.7. Intestine

Human in vitro models of the intestine are of crucial importance in pharmacokinetics studies. However, such conventional prototypes fail to recap the physiological microenvironments (e.g., cyclic peristaltic motion) of the human gut. Thus, microfluidic technology provides a powerful in vitro platform of the intestine to emulate an in vivo-like microenvironment for drug testing and various microphysiological studies. For instance, drugs taken via the oral pathway have to transverse the small intestine barrier to reach the bloodstream. Villi are the salient bridge-key to absorption and their physio-morphology must be maintained on the chip [138]. In this field, 2008 was the year when Kimura and collaborators [139] designed a microfluidic system consisting of two lumens separated by a porous membrane. A stirring pump was incorporated into the device in order to control the fluid flow in the system and to create a dynamic environment for the cells. Caco-2 cells were cultured in the system for over 30 days and the fluorescent dye, Rhodamine 123, was added into the system to investigate the cell permeability. Subsequently, the team of Imura [140] designed specific intestine-on-chips to mimic the intestinal system. The latter consisted of glass-slide permeable membrane and a PDMS sheet containing the channels. Caco-2 cells were cultured on the chips. The first 3D hydrogel structure was developed by Sung and coworkers [141]. These pioneers were able to design a specific device emulating the human intestinal villi. This step has been followed by a tremendous advancement in the world of gut-on-a-chip technology by Kim and his team [142], with the production of bionic devices. In fact, the recreated microenvironment of the intestine was performed via shear force and cyclic strains. Caco-2 cells demonstrated a prolonged growth rate by maintaining the microbial flora within the human intestine.

This state-of-the-art microfluidic gut-on-a-chip platform designed by Kim and Ingber [143] supplies in vivo-like peristaltic movements, and fluid dynamics were applied to the entire system to induce the differentiation of multiple intestinal cell types. The astonishing characteristic of this device was the ability to recapitulate the 3D structures and complex physiological functions of the human intestine. That led to the development of an intestinal inflammation model on a gut-on-a-chip platform. The latter showed promising potential in terms of use for the pathophysiological investigation of human intestinal inflammation produced by the overgrowth of bacteria [144].

The morphology, physiology and complex structure of the human intestine pro-vided a specific platform for drug screening, reflecting the salient role of the intestine in terms of diverse parameters, encompassing the intestinal microbiome, inflammatory cells and peristaltic-related mechanical deformation during intestinal ailments [144]. This system authorized the exploration of the etiology of intestinal disease and identified therapeutic targets and drugs. This investigation revealed the potential of intestine-on-a-chip systems for personalized medicine and disease modeling studies on intestinal cells. The latter were cultured alone or with endothelial cells, including HUVECs [142]. Genome fidelity was low that the chips highly emulated intestinal function.

Two pilot studies conducted by Kasendra et al. [145], and Vandussen et al. [146] where intestinal tissue microengineering and state-of-the-art organ-on-a-chip technology were combined in order to establish in vitro biological models of the human duodenum. It is relevant to note that the intestinal epithelial cells cultured in the chip were obtained from endoscopic biopsies or organ resections. This chip represented the closest model to the living human duodenum and reproduced crucial traits of the small intestine. Similarly, the incessant advancement in this field gave birth, in 2019, to a gut-on-a-chip microdevice that succeeded in providing mechanical movements and the injection of continuous fluid flow into the microfluidic device to investigate the role of physical stimulus on intestinal morphogenesis. This gut-on-a-chip platform has successfully ensured the culture of human intestinal Caco-2 and primary intestinal epithelial cells. The advantages of this biochip include the incorporation of mesenchymal cells and other types of cells, which highlight its potential contribution to intestinal morphogenesis. This model led to a tri-dimensional morphology, reflecting a perfect match with the related computational simulations. Recent findings in this field underscored our knowledge of the intestinal microbiome [147] and intestinal physio-morphology [148].

### 4.8. Uterus

As a complex ecosystem, the female reproductive tract contains the uterus, ovaries, fallopian tubes and cervix. These tissues and organs are directly implicated in the secretion of sex hormones, production of ova and maintenance of pregnancy during the gestation period [149]. The reconstruction of a total uterine intricate system on a micro-architecture biochip is of great impact for testing and assessing the efficacy and/or toxicity of new drugs [149], especially for in vitro fertilization-embryo transplantation (IVF-ET) [150]. In the same line, a diverse range of researchers have tried to reconstitute the physiological function of the reproductive system [150,151]. Accordingly, an extremely advanced and promising microfluidic device called EVATAR (‘avatar’, the digital representation of an individual, plus the name ‘Eve’) has been devised by Xiao and his team [149]. Such a masterpiece represented a powerful tool for drawing phenocopies of the human menstrual cycle and pregnancy via reproducing the endocrine loops between organ modules for the ovary, fallopian tube, uterus, cervix and liver, with a sustained circulating flow between all tissues.

### 4.9. Vessels

The blood vascular system consists of an outstanding intricate network of blood vessels in terms of arteries, arterioles, capillaries, and veins that convey blood from one organ to another, ensuring the correct functionality of every single organ. Hence, blood vessels represent the elementary and vital building blocks of this system [152] since they deliver nutrients and oxygen to all the organism tissues and remove waste products. Several studies succeeded in the setup of blood vessels on miniaturized devices [61,63]. According to Zheng et al. [153], morphogenesis and the development of mechanical stimulation, as crucial parameters, are needed to provide a miniaturized microdevice capable of delivering fluid shear stress (FSS) and cyclic stretch (CS) simultaneously or independently, in order to correctly mimic the vascular physiology. Additionally, the reconstruction of vessels-on-a-chip, as a complex task to achieve, is not only useful to connect each other to the various compartments of a body-on-a-chip application [152], but it also allows us to closely examine system dysfunctions caused by specific ailments, e.g., angiogenesis, in patients with tumors, diabetes or with wounds, or to investigate the effects of inflammation on vascular integrity or interrupted flow (e.g., thrombosis) [154].

### 4.10. Tumor

Tumor-on-a-chip represents another interesting area of organ-on-a-chip scientific investigations. This technology holds enormous potential for the discovery of new cancer therapeutics that target cancer cells with more specific precision. According to Shuler [155], the tumor-on-a-chip systems can be categorized into three major subtypes, which are firmly dependent on the purpose of the experimental biochip; (*i*) monitoring anti-angiogenesis, vascularization, and migration of cancer cells; (*ii*) screening and assessment of the effects of drugs and/or nanodrugs on tumor cells, and (*iii*) detection of blood-borne cancer markers. In the field of carcinology and cancer therapy, microfluidics science has tremendously impacted this area of investigation, especially in terms of the creation of size-tunable 3D tumor spheroids, sorting of tumor cells, and balancing of cell density in co-cultures. Recently, new 3D tumor-on-a-chip microfluidic devices have been devised in order to explore the inter-communication, also known as ‘crosstalk’, between normal cells and cancer cells. Likewise, as a state-of-the-art technology, microfluidics has opened the gate to developing new targeted drug delivery systems [156]. Such sophisticated miniaturized architectures have also allowed scientists to collect and detect circulating tumor cells (CTCs), leading to an obvious amelioration of cancer diagnosis and tumor marker detection in the early stages. It is relevant to know that numerous nanoparticle-based screening systems have been conceived in order to mimic the microenvironment of tumor cells and adjacent vessels, leading to a significant improvement of drug delivery and drug screening [157]. Recently, strategies for studying tumor neovascularization have received nonstop interest. For that reason, a new generation of microfluidic capillaries has been massively investigated and developed to shed light on the interconnection between tumor cells and blood vessels [158]. Such microfluidic capillaries were based on three channels, encompassing the central channel (for seeding ECs) and side channels (for seeding tumor cells). The above-mentioned channels were linked to each other by filled gelatin pathways. Zervantonakis et al. [158] succeeded in seeding tumor cells on the tumor-vessel co-culture micro-architecture devices. Such a result led to an obvious accumulation of tumor cells in the adjacent chamber to the vessels, reflecting an important contribution to the investigative reports regarding the transport and invasion mechanism. This tumor-on-a-chip platform represents the first tremendous step to exploring how tumor cells grow in tissues. For the record, a wide range of tumor-on-a-chip systems have been used for almost all the tumor types, encompassing breast cancer, bone marrow, brain, intestine, lung, liver, and even urinary tract cancer.

## 5. Organs-on-Chips Technology: Almost Everywhere

Such revolutionary technology has widely impacted the research field, especially in areas such drug screening [159], disease modeling [160], cancer cell migration [161,162], axon growth [163], neuronal models [164,165], single-cell analysis [166], cell–cell interaction and cell-ECM studies [167], cell co-cultures [168], and even fluid gradient-involving studies such as bacterial chemotaxis [169]. Herein, we discuss the presence of such cutting-edge technology in nearly all the fields of scientific research and pre-clinical investigations.

### 5.1. Biological Mechanism Investigations

The coupling of OOC technology and microfluidic science has highly contributed to the study of the structure and function of specific organs, and even at a more accurate scale, these two state-of-the-art disciplines investigated the interactions between tissues and organs [170]. For instance, Zhang et al. [171] designed an elementary ‘human-on-a-chip’ with the purpose of studying the regulating ability of Transforming Growth Factor (TGF)-b1 with the introduction of a four-tissue/organ system involving liver, lung, kidney and adipose tissue. Such a masterpiece impersonated the real human microenvironment in vitro. Similarly, Huh et al. [18] were the pioneers who built the first ‘organ-on-a-chip’ micro-model, allowing them to reconstitute the lung function with cyclic mechanical strains of the alveolar capillary interface. This biochip permitted the increase of the uptake of external substances into the epithelial and endothelial cells, leading to a smooth transportation of these substances to the microvascular chamber, reflecting a high similarity with the real steps taking place within a real lung alveolus. Such a model has ensured a significant increase in nanoparticle absorption once the lung alveolus interface was exposed to mechanical stress. It is relevant to note that regardless of the reaction of organs and tissues to the external environment, the inter-specific interactions between organs also can be accurately investigated. In the same line, Sances et al. [172] established the auxiliary action of brain microvascular endothelial cells in the maturation of human neurons via co-culturing these two cell types on a biochip. Similarly, in 2017, Skardal and coworkers [173] fabricated a multi-organ-chip platform considering the inter-organ responses, to obtain a more accurate therapeutic reaction. Further details about the biological mechanism studies of OOC technology will be discussed in the next sections.

### 5.2. Regenerative Medicine

The reconstruction of organs seems to be crucial in the field of regenerative medicine [174], reflecting the tremendous potential to cure or replace damaged tissues and organs without the existing complications of using organ allografts. The perfect combination between microfluidics and OOC technology helped to unveil key features of different methodologies of biochip fabrication and how they impact cell viability and tissue functionality in the context of regenerative medicine. Within this framework, Guo et al. [175] conducted a pilot study in which they were able to recover a neuron on a microfluidic chip before the emergence of the organs-on-chips trend. Likewise, Tang et al. [176] were able to quantify the orientational regeneration of injured neurons by natural product concentration gradients in a 3D microfluidic device. In harmony with this field, 3D bioprinting and electrospinning technologies have highly impacted regenerative medicine [177], particularly in the construction of a micro-architectures heart-on-a-chip model with endothelialized human myocardium with the purpose of substituting an unhealthy heart [89]. In the field of stem cells, OOC technology has showed a high potential in terms of ensuring their culture and differentiation. Of note, the targeted differentiation of stem cells has generated high prospects, especially in terms of organ regeneration [178,179]. In 2012, Park and his team members [180] developed a miniaturized device as a primary organ chip with the purpose of investigating the different osteo-genic capacities of human bone marrow- and adipose tissue-derived mesenchymal stem cells. Musah et al. [32] led another research study in which they succeeded in divulging the differentiation of iPSCs to functional human podocytes, leading to the regeneration of the kidney glomerular-capillary-wall function on-chip.

### 5.3. Drug Discovery and Toxicity Assessment

During the last decade, as OOC technology has been in exponential development, correlated platforms and micro-architecture systems have been increasingly used for drug discovery, drug screening and delivery. In fact, a heart-on-a-chip design fabricated by Agarwal and collaborators [181] summarized the curative effect of isoproterenol. As well, kidney-on-a-chip [79], liver-on-a-chip [182], intestine-on-a-chip [183] and many other types of organ-on-chips were used to test substance toxicity and to screen drugs. Additionally, an astonishing multi-organ-chip platform integrating the intestine, liver, skin and kidney was created in 2015 by Maschmeyer and his team [184], in order to test repeated-dose systemic toxicity. Recently, researchers succeeded in developing multi-organ-chips to identify anti-angiogenic and anti-tumor drugs [185] and to reveal the anticancer activity of a flavonoid luteolin with integrated liver and cancer tissues [186].

### 5.4. Platforms for Ailments and Carcinology

The OOC, as a state-of-the-art technology, provides a marvelous platform for disease modeling, such as the case of pulmonary edema [187], protein-induced lung inflammation [188], central nervous system (CNS) disease [189] and type 2 diabetes (T2D) [190]. Within this field, a pilot investigation was conducted by Benam et al. [191], in which a chronic obstructive pulmonary disease was studied on a biochip, with the identification of new biomarkers of disease progression. Moving forward with disease modeling, several scientific works have intensively investigated the application of blood–brain barrier (BBB)-on-chip platforms in order to study the effect of inflammation on barrier function, and how to interfere with this process [102,192,193,194].

According to Fitzmaurice et al. [195], cancer has the second largest risk of death, with a high global fatality rate; therefore, tumor and cancer biology represent a major area of interest to study in OOC platforms. In 2015, Fan et al. [196] and Ling et al. [197] performed two investigations where they designed two in vitro sophisticated 3D tumor microdevices with spheroid shapes, on the basis of the bioprinting technology. In lung cancer, Hassell and his group members [198] succeeded in the recreation of a masterpiece lung-on-a-chip with non-small-cell lung cancer. Such a biochip permitted the researchers to examine and follow the growth of tumor cells in a controlled microenvironment. They were also able to analyze tumor dormancy and the response to tyrosine kinase inhibitors used therapeutically, as clinically relevant processes. It was found that the signal transduction of epidermal growth factor receptor and MET protein kinase may affect the sensitivity [198]. Of note, different OOC micro-models have also been designed to screen anti-cancer drugs in a breast ductal carcinoma in situ device [199]. They highly contributed to investigations of the mechanisms of metastasis of breast cancer cells using an in vitro 3D bone-on-a-chip device [200]. Within this context, Table 3 lists the main disease models, the associated chips and their relevant potentialities.

### 5.5. Radiobiology

Radiobiology, as a new area of biomedical investigations, has appealed to OOC technology. Snyder and his team [266] were the pioneers who succeeded in the design of a microfluidic chip with liver tissue. Such a biochip enabled this group to validate the liver tissue injury from space-like radiation. It also helped in the assessment of pro-drug amifostine treatment and its effects. Subsequently, Torisawa et al., with a bone-marrow-on-a-chip device, were able to corroborate the direct impact of γ-radiation on the hematopoietic potential of bone marrow. Such a sophisticated biochip was the model of choice to substantiate the beneficial effects of Granulocyte-Colony Stimulating Factor (G-CSF) and bactericidal/permeability-increasing protein (BPI) to healing from radiation exposition separately in 2014 and 2016 [267,268]. Likewise, a gut-on-chip model, developed by Jalili-Firoozinezhad et al. [244], was exposed to radiations. The investigation revealed that γ-ray radiation led to critical damage at a large scale, particularly in terms of the aggravation of the generation of reactive oxygen species (ROS), augmentation of cytotoxicity, apoptosis and DNA fragmentation. Critical damage of the tight junctions and integrity of the intestinal barrier occurred with a significant loss of the villus-like normal morphology. Accordingly, the use of dimethyloxaloylglycine (DMOG) within this miniaturized device has been shown to effectively stop the aforementioned damage caused by radiation. To sum up, though the OOC technology has not been broadly used by clinicians, it disclosed relevant success potentials in investigations of disease modeling, drug screening, personalized medicine, disease mechanisms and even experimental regeneration medicine. Such success in pharmacological trials has moved the OOC technology to the next level of investigation, thereby touching the field of radiobiology, particularly via enabling the development of protective therapies after radiation exposure and by stimulating the optimization of personal radiotherapy.

## 6. Human-on-a-Chip: Reality vs. Myth?

During the past decade, OOC technology, as ‘a fetus’, has gradually matured, especially in terms of the advancement in disease modeling and the establishment of multi- organ-on-a-chip. Despite this astonishing progress in the world of OOC, it seemed insufficient to rely on an emulated single-organ model for a comprehensive understanding due to the highly complex and intricate interactions between human organs. According to Lee and Sung [269], single-organ biochips greatly fail to entirely reflect the complexity, functional variations and integrity of an organ’s accurate role. That is why a new concept has emerged, also known as ‘multi-organ-on-a-chip’, otherwise referred to as ‘human-on-a-chip’ or even ‘body-on-a-chip’ [270].

In 2004, a revolutionary year in biomedical investigation, Dr. Shuler and his team were the pioneers who proposed the concept of reproducing human physiological functions in chip devices [271]. Recently, the nonstop growing demand for in vitro models and chips integrating multiple organs has become a major topic, reflecting the salient multifaceted step forward in OOC technology.

Currently, several investigations are deeply working on developing multifaceted chips capable of representing multiple organs in an integrated manner with an accurate emulation of human tissue [272,273,274,275]. Whilst the multi-organs-on-a-chip concept remains in its premature infancy, major breakthroughs have been made, encompassing the design of two organs [276,277], three organs [173,278], four organs [184,279] and even ten organs on the microfluidic chip [280].

In the world of drug delivery, a new biochip has been designed for the study of physiological pharmacokinetics (PKs) and pharmacodynamics (PDs). It successfully allowed the prediction of clinical patient data of cisplatin PDs. Herland et al. [281] demonstrated that this model is firmly connected through coupled micro-vascularized organ-chips, with the purpose of investigating the PKs and PDs parameters of oral and injectable medicines. In the same year, Novak and colleagues [282] showed that experimental trials with this particular microdevice achieved an automated system. The latter was based on the robotic microfluidic coupling of multiple-organ chips and maintained long-term cultures of organ-specific functions for three weeks. Such an automated multi-organ chip system has the potential to ameliorate the prediction of drugs at different levels, especially in terms of absorption, delivery, metabolism, excretion and even toxicity assessment for clinical trials. In harmony with these findings, Oleaga et al. [279] unveiled that the microdevice was mainly based on four types of tissue—heart, liver, muscle, and neurons. As aforementioned, such a sophisticated model contained a heart (as the salient organ in the human body), a liver (playing the role of the body’s most important filter, particularly when drugs and medicines are applied), a skeletal muscle (as the organ responsible for glucose storage in the body), and neurons (highly sensitive cells). A period of 14 days was critical to achieve the complete feasibility and functionality of this system. Of note, cells used in the biosystem were primary cells and cells derived from human-induced pluripotent stem cell (iPSCs). The measure of the heart rate, muscle contractility, neuro-electrophysiology, and production of liver albumin and urea were the major outcomes, characterizing a sophisticated model with the purpose of predicting and assessing toxicity in a multiple-human-organs-chip. In 2015, Maschmeyer et al. [184] succeeded in designing a complex model integrating a pre-formed bowel and skin models into a hepatic spheroid and renal epithelial barrier tissue model. The establishment of such a miniaturized model was of major importance, especially in terms of supporting the functions of four different types of organs in a co-culture over a period of exceeding one month, reflecting a unique masterpiece essentially based on a structure using the simulated physiological fluid and tissue environment of the human body. Such a chip highly mimicked the key steps in the life cycle of a drug. Firstly, there was the step of absorption, followed by its metabolism in the small intestine, then the drug was metabolized by the liver, and the final step was its excretion by the kidneys, summing up the main stages that determine the efficacy and safety of drug treatments. Such multifaceted systems represented the first brick in the pyramid of the reconstitution of complex microenvironments, simulating multifaceted reactions and interactions between tissues, to investigate drug toxicity testing, toxicological screening and assessment at a metabolic, molecular and genetic scale, and to ensure the construction of organ-on-a-chip sophisticated models as the ultimate goal.

This step into the future of biomedical investigation concurrently permits the reconstruction of multiple organs and makes researchers salivate for a sophisticated, attractive new field. The establishment of such miniaturized intricate platforms requires different cell types and tissues, which should be concomitantly connected by channels (bionic blood vessels [63]), ensuring the integration of multi-organs, and an accurate examination of their interactions [275,283]. Such multi-organ micro-architectures can be separated into static, semi-static and flexible systems [284]. Regarding static multiple organs, the latter are well organized into single connected systems. Within semi-static models, the organs are joined through fluidic microchannel intricate networks with Transwell^®^-based tissue inserts [133]. Concerning the flexible device, Rogal et al. [284] unveiled that flexible microchannels are used to interconnect individual organ-specific platforms. Such flexibility has highly facilitated the recreation of multiple organs. During the last two decades, advances in OOC technology have basically been linked to four main parameters: design, modeling, manufacturability, and usability. In 2018, an inventive combination of laser technologies was established by Díaz-Lantada et al. [285]. Such accurate and sophisticated art-of-state technologies are feasible for mass-produced chips with holding utility for energy, transportation and aerospace industries.

To wrap up, the expectation of integrating multi-organ-on-chips to replace the insufficiency of conventional in vitro models seems to be realized. Owing to the astonishing potential of these miniaturized platforms to impersonate the micro-physiological structure of internal organs and their intricate interactions with diverse soluble substances and metabolites, investigating the impact of air pollution on the body, the early development of drugs, and even studying interactive effects between organs in vitro has come to light. Table 4 describes the multi-connected organs-on-a-chip and their potentialities in drug development investigations. Nevertheless, current available multi-organ chip models and systems are mainly used for the systemic processes of oral and injectable drugs, but models for investigating drug delivery and even cytotoxicity testing and assessment are still suffering from scarcity.

## 7. Human Body-on-a-Chip Platforms: Is There Any Limitations?

Living animal models have served for a long period for diverse experimental tests, physiological processes, disease states, molecular mechanisms, accurate phenotypic and genetic studies, and thus, they have reflected the mainstay of scientific investigation in institutes of research and pharmaceutical companies. However, a blocking limitation has emerged on the surface in terms of preclinical animal experiments that often do not genuinely imitate the pathophysiological traits and functions of human tissues and organs, leading to imminent failure in the prediction of therapeutic responses in human clinical trials. Whilst OOC technology is still incapable of entirely replacing conventional animal studies, it seems actually able to bridge the gap between animal experimental tests and clinical trials. A wide range of pros and potentials of this state-of-the-art technology, encompassing increasing the quality and accuracy of results, decreasing the manufacture and maintenance costs of on-chip devices and systems, and absence of any cruelty towards animals, has led to an intensive encouragement of the scientific committee to move forward using this sophisticated body-on-a-chip system to refine, reduce, and eventually replace animal models.

Multi-organ-on-a-chip platforms, particularly human-on-chip platforms, can be utilized for myriad applications, from disease modeling to personalized medicine passing through preclinical drug testing. This sophisticated microfluidic system has shown high potentialities in terms of shortening, refining, and/or minimizing the need for early clinical experiments in the near future while providing more efficient treatment regimens. Thus, such technology enables, at the current time, accurate investigations on vulnerable subjects that are excluded from clinical trials, including children and/or pregnant women. During the last few years, OOC technology has shown new promising horizons in terms of disease modeling and treatments that connect multiple organs at once, such as the case of cancer metastasis or radiotherapy, as well as cross-organ interactions and systemic effects. The insertion of patient-derived biological materials, such as metabolites, cells and microbiome on different human-on-chip platforms makes diverse processes possible, encompassing the mimicking of rare ailments, repurposing of orphan drugs, reduction of the risks of false-negative and false-positive readouts and the evaluation of undesirable drug effects or drug–drug interactions.

Conventional culture systems are well characterized by changing the cell medium in a regular way at precise intervals, leading to a nutrient and/or pH shock to the cells, reflecting a blockage in terms of endogenous autocrine or paracrine signaling processes that occur in vivo [288]. Or, as for the OOC technology, the latter ensured an entire dynamic and continuous sequential fluid flow into the cell cultures and between organs and therefore can control the proper spatiotemporal presentation of autocrine and paracrine cell-secreted signals and dissect their role in the behavior of stem cells. In the field of vaccination and immunotherapeutic drugs, Shanti and coworkers [289] demonstrated that both circulating, and tissue-resident immune cells can be added to entire system in order to investigate vaccines and immunotherapeutic medicines in a patient-specific manner, and this operation has been progressed via the association of fluidically linked endothelial lumen of body-on-chip systems with the high advancement in immune organ chips. In the realm of immunity and its crucial role in various ailments, Moore et al. [290] showed that the integration of different types of immune cells in human-on-chip systems represents an astonishing advancement towards the development of more accurate predictions for drug discovery, drug delivery and precision medicine.

As with every single emerging technology, human body-on-chip platforms have revealed several limitations. For instance, the high in vivo relevance of these intricate and sophisticated platforms comes at the price of low throughput, where only a few replicates can be performed at once. According to Ingber [291], the organ chips may exhibit considerable variation and inconsistency between different manufactured batches, different laboratories, as well as different users. It seems also so much simpler and easier to use multiplex organoid cultures, transwell-based microphysiological systems, or even simple microfluidic devices in order to produce findings more quickly and at lower cost during the early stages of drug discovery and drug delivery processes, which are likely to be more appealing to the pharmaceutical industry. It is pertinent to note that setting up multiple parallel experimental trials on a human-body-on-a-chip device represents a real challenge, requiring highly trained personnel specialized in different single-organ models. According to Ewart and Roth [292], other various limitations can be enumerated, such as the high cost of microfluidic technology and OOC platforms and the limited availability of several reagents, materials, cell lines, and equipment. Additionally, to add the list mentioned above, a critical burden has emerged in terms of scaling up the manufacture of devices and platforms in a reproducible and robust manner across different preclinical and clinical laboratories to an industrial pace. State-of-the-art devices of micro-architectured, miniaturized, automated, yet intricate multi-organ-on-chips can, however, boost throughput and the number of replicates per platform and address some of the medical and pharmaceutical challenges at a large scale as well. In 2018, Novak and his group [293] revealed that gathering research laboratories and startup companies together has led to an outstanding progress and advancement in terms of mass production of chips and the design of user-friendly automated chip culture systems, reflecting an expansion of culture human-body-on-chip platforms in the near future. Though the cost of a single commercial chip can be as high as several standard 2D/3D culture systems, more reasonably priced chips are being introduced every single day.

The choice of cell source and the culture microenvironment represented a critical burden at the moment of designing OOC platforms and devices. For those reasons, cancerous cell lines are massively used in this technology. In fact, immortalized cell lines can be indefinitely cultured in vitro, tending to accumulate karyotypic aberrations that can lead to relevant differences in terms of drug responses. In the same line, primary cells played a key role as astonishing and promising alternatives to commercial cell lines that are able to retain functional and metabolic properties similar to those displayed in the original tissues [145]. This type of cell is often obtained from tissue biopsies collected for diagnostic purposes and can express pathological phenotypes. Additionally, they are so demanding in terms of specialized medium, specific ingredients and culture preparations. They also have limited availability, and can display donor or batch variations (genetic, epigenetic, morphological, or functional). According to Liu et al. [294], induced pluripotent stem cells, also known as iPSCs or adult stem cells, from tissue organoids have emerged as a solution to overcome some of these challenges, revealing a high potential in terms of patient- and disease-specific cell lines that can be virtually differentiated into any cell type. Nevertheless, the long-term efficacy and large-scale manufacture with high reproducibility of iPSCs is still uncertain and is yet to be confirmed. Until now, scientists and researchers have been nonstop working to design efficient approaches and protocols with the purpose of differentiating iPSCs into any type of cells that fully resemble the original tissue [295].

Another limitation has been associated to the PDMS as the major material used in the design of chips. From a bioengineering point of view, the biomaterials used can also have effects on the performance of the manufactured biochips. It showed several drawbacks particularly in terms of absorption of small molecules and adversely affects drug screening and pharmacokinetics/dynamics (PK/PD) modeling [296]. In harmony with this, another study showed the high adsorptive level of proteins on the surface of PDMS, which results in the given drug or stimulating substance not fully interacting with the cells within the chip [297]. Moreover, the interaction between the cells and extracellular matrix (ECM) is pivotal for organ function as well as pathogen colonization; though, synthetic biomaterials are typically characterized by mechanical properties highly dissimilar to native ECM [298]. Although in silico modeling can circumvent this limitation to some degree, other biocompatible, transparent, and oxygen permeable polymers could provide a better template for disease modeling, drug toxicity assessment and personalized medicine studies. Various materials, such as poly(methyl methacrylate) (PMMA), the recently reported polymer that offers promising outcomes in avoiding the absorption of small molecules and is impervious to small molecules, reflects a more reliable approach compared with PDMS. In 2019, Nguyen and collaborators [299] led a pilot study in which they succeeded to bond PMMA polymer to polyethylene terephthalate (PET) track-etched membranes within microfluidic devices, and thus, it enabled the assessment of the cytotoxicity of vincristine on human lung adenocarcinoma cells. Otherwise, it is pertinent to know that natural and/or synthetic hydrogels and/or decellularized ECM can be incorporated into chip micro-architecture to promote tissue growth and enable the creation of complex 3D structures. These materials can be designed to degrade along with the tissue growth and enable tissue–tissue interactions within the chips similar those observed in vivo. Whereas patient-specificity represents an amazing feature of organ chips, patient tissue cells are usually limited in number or exhibit low proliferation, and their collection may require invasive techniques. The time factor has also been indicated as a potential limiting factor for organ chips [300].

Amongst the limitations of the OOC technology, culture time seems to be a critical factor particularly restricting the study of the long-term effects of disease and its progress during a long period, such as the case of chronic hepatitis. Likewise, Wnorowski et al. [301] revealed that wide acceptance of this technology by the scientific committee is still oscillating between approval and rejection. In the same line, the absence of a unique universal device design, the lack of appropriate validation and quality control of organ-chips, and the need to prove their robustness and reproducibility, before achieving a translational step, represent another range of hurdles facing such a technology [302].

To sum up, organo-mimetic devices usually require operational specific equipment ensuring fluid flow through specific microtubing and applying shear stress to the cells under dynamic conditions (e.g., pressure), reflecting critical difficulties in terms of cost and adaptation [303]. We recapitulate in Table 5 the main pros and cons of this technology, reflecting a terrific masterpiece of technological and biomedical advancements in terms of applications, biological sampling, material of fabrication, and even the miniaturized size, with minor flows to circumvent in the near future.

## 8. Biosensors in Organs-on-a-Chip Platforms

How can a biosensor be described? According to the International Union of Pure and Applied Chemists (IUPAC), a biosensor is ‘a device that uses specific biochemical reactions mediated by isolated enzymes, immunosystems, tissues, organelles or whole cells to detect chemical compounds usually by electrical, thermal or optical signals’ [304]. Two main aspects can be deducted from the aforementioned description: the biorecognition material (i.e., antibody, aptamer or enzyme) where the biosensor can biologically detect the analyte with high specificity and selectivity. A first step which is followed by the transducer (i.e., optical, electrochemical, mechanical) that ensures the transformation of the biorecognition event into a physical quantity. The latter can be measured and analyzed by an electronic tool that monitors the results for the final user [305]. Recently, there was an upsurge in biosensors for nonmedical fields, notably for the development of cell culture systems. Such a research sector has witnessed the conceptualization of various biosensors. Amongst the list, electrochemical biosensors are still the most relevant due to their high-throughput quantification and analysis of biochemical interactions. Nevertheless, optical biosensors could not be underestimated, especially in terms of their emergence over recent years. This rise in optical biosensing technology is firmly associated with a wide analytical coverage [306]. Integration and high-throughput analysis represent key elements for succeeding in the development of relevant biosensor devices for different organ-on-a-chip platforms. Within this context, microfluidics seems to be indispensable to provide simultaneous analysis and assure low sample and reagent consumption. Over the last two decades, several hurdles have limited the integration of biosensing systems for in situ purposes such as the biodetection of segregated biomarkers from OOC devices. It is pertinent to note that the high availability of commercial electrodes has steadily helped the development of electrochemical biosensors, which represent a crucial component of current biosensing systems. To wrap up, the integration of optical and electrochemical biosensing systems within microfluidic chips seems to be pivotal in order to monitor essential organ functions, encompassing organ activity such as cardiac beating (e.g., cantilevers and MEAs (multielectrode arrays)), metabolic parameters (such as porphyrin-based oxygen sensing, amperometry, and voltammetry); barrier integrity (TEER/ECIS) (Transepithelial electrical resistance/electric cell impedance); and biomarker secretion (e.g., amperometry coupled with biorecognition elements).

Table 6 describes the most relevant optical- and electrochemical-based biosensing devices, with the detected biomarker(s) and the detection threshold.

## 9. Hydrogels in Organs-on-a-Chip Engineering

The reconstitution of a human body-on-a-chip represents the ultimate goal of OOC technology. Such a purpose has been conceived in order to replace animal models in preclinical drug testing and accelerate the process of drug discovery. Recently, a paradigm shift in tissue bioengineering and biomedical investigation towards constructing 3D human tissue/organ models in vitro has emerged to develop accurate diagnostics and treatment substitutes. In fact, materials used in the fabrication of OOC devices include glass and polymeric materials, mainly PDMS material. The latter has enabled real-time and high-resolution imaging due to their optical transparency and gas permeability. Nonetheless, such a material has several limitations. Amongst the list, absorption of small hydrophobic molecules or drugs from microfluidic solutions seems to be the most limiting hurdle, notably in cellular experiments, which may critically modify the determination of drug dose response [2]. To surpass such hurdles, more adequate material substitutes have been designed. Currently, hydrogels seem to be the most promising alternative material for cell growth, with the specific attributes of high biocompatibility, permeability and tunable physiochemical properties. As hydrophilic biomaterials, hydrogels have been applied in OOC microfluidic systems to render PDMS nonabsorbent because they are less drug-absorbent than PDMS. Additionally, the use of such material in OOC models has permitted the formation of vascularized networks, tissue–tissue barriers, and parenchymal tissues. In the same line, Table 7 describes examples of different types of hydrogel-based OOC platforms.

## 10. Organ-on-a-Chip Platforms: A Glance on the Market

The adoption of OOC technology by the committee of clinicians and the pharmaceutical industry urgently needs to overcome several challenges and obstacles [29,323,324,325]. Thus, in the past few years, the fever of start-ups in the field of innovative biomedical technologies has emerged and drowned the market. The latter has contained a mosaic of start-ups that are more specialized in developing specific organ-on-chips, while others propose developing an independent biochip with the possibility of developing different organs, intend to manufacture a single-organ device, and or prefer multi-organ platforms to assess interactions between organs. In this field, every single player recommends its own unique technology, with specific applications, pros and cons. Despite all these advancements and tremendous state-of-the-art technologies, the gap is still present between market needs and available technologies, reflecting the urgent need for new leaders in this field to show up and demonstrate the predictive validity and reproducibility of OOC platforms for disease modeling, drug discovery and personalized medicine. According to Roberts et al. [326], the implementation of such technology by industrials will start with contract research organizations (CROs), which support biotechnology and pharmaceutical industries in R&D services.

Ma et al. [115] reported the interest of different countries around the globe towards OOC technology and how governments finance projects directly linked to this technology. For instance, in the European zone, several academic structures and research centers initiated an open project entitled Organ-on-Chip in Development ‘ORCHID’ in 2017, which gave birth to the European Organ-on-Chip Society, also known as ‘EUROoCS’. Such a structure facilitated (and still does) the collaboration network across academic, research, industrial, and legislative institutions and agencies. In the American continent, the US federal agencies including the National Institute of Health (NIH), National Science Foundation (NSF), and Department of Defense provide different seed funds by the means of the Small Business Innovation Research and Small Business Technology Transfer programs. Such funds targeted accurate research in the field of OOC technologies, particularly in terms of their development, standardization, and commercialization for use in the drug development sector. Regarding the Asia-Pacific zone, mainly China, Singapore, South Korea, and Japan, this region is deemed to be the emerging market, owing to government support for healthcare technologies. For instance, in 2018, the Chinese Academy of Science launched a 5-year initiative of ‘Organ Reconstruction and Manufacturing’. Such an important investment allowed (and still does) the nonstop progress of this cutting-edge technology with a reduction in its whole cost of fabrication, standardization and establishment.

In terms of companies, few have already started investing in developing certain OOC systems and devices. Project funding, collaborators and partnerships are constantly increasing, underscoring the commercialization potential of the OOC realm [327]. Emulate Inc., CN Bio Innovations, Mimetas BV, InSphero AG, Ascendance Biotechnology, Inc., Kirkstall, Hurel Corporation, SynVivo, AxoSim Technologies LLC, Nortis Inc, Orga-novo Holdings, Inc, Tara Biosystems, Elveflow, TissUse GmbH, and Roche Holding AG represent the most featuring leaders in the OOC industry. They are constantly in competition to take over the market space of the best OOC models for disease modelling, drug discovery and testing and personalized medicine. Consequently, drastic quantitative and qualitative investigations are highly utilized by OOC manufacturers in order to prove that these diverse OOC platforms represent living mimics of human organ functions and to accelerate their adoption by CROs as well as by industrials and health care systems [323]. Table 8 sums up a number of pioneers in commercialized OOC devices and platforms. The Reference of Zhang and Radisic [323] is an excellent article for interested readers for extensive details and discussion in this regard.

## 11. What Is and/or Who Is Next on the Chip?

During the last two decades, the applications of organ-on-a-chip technology were constantly expanding, enabling the study and the analysis of a wider range of infectious diseases, critical ailments and antimicrobial discoveries, particularly via overcoming the technical hurdles of current technologies. Within this context, Barr et al. [329] were the pioneers who designed a mucus-producing lung-on-chip model, mimicking a mucosal surface with constant fluid flow and mucin secretion dynamics, in order to investigate the trilateral interactions between bacteria, bacteriophages and mucosal epithelium. This team also analyzed phage adherence to the mucus layer. Hence, this model enabled them to establish a non-host-derived layer of immunity against microbial infections. Of note, bacteriophages have been used for their antimicrobial potential, but the majority of the therapeutic validation has been accomplished in animal models. As a promising tool in biomedical investigation and biotherapeutics, diverse OOC platforms are offering unprecedented grounds for the validation of phage therapy approaches within organs of interest, leading to an ability to track the emergence of any kind of phage resistance. Additionally, regarding gut and/or intestine microfluidic models, the latter seem to be terrific. Thus, we need to determine how to afford more realistic microenvironment with the purpose of studying the gut phagosome, phage-bacteria interactions, and tracking phage adaptation [330]. It is important to notice that biochips are also acquiescent to the introduction of genetically modified phages and bacteria for real-time tracking via the insertion of fluorescence markers or CRISPR locus for the quantification of target populations, thereby rendering them valuable tools for evolutionary science.

### Worms-on-a-Chip ‘WoC’

From the past to the present, humans have long been inspired by nature in all features. Despite the nonstop advancement in technology, the diversity and complexity of natural species remain second to none. As compared with plentiful biomimetic artifacts to date, nature-born life may not be stupendous just in a single field. Nevertheless, they are more robust and flexible to deal with multiplexed data in the real world. Therefore, though laboratory-grown tissues still suffer from expressing full functions in terms of biological responses and interactions, the tiny nematode can certainly supply more comprehensive functionality than its counterparts. Unlike other higher animals, *C. elegans* represents an easy model in terms of being small in size, easy to maintain, low in cost, and sophisticated in functions. It has been widely used in biomedical investigations, including for air quality monitoring [331]. That is why researchers went further with a multi-cellular living model when they attempted to incorporate *C. elegans* into different chip platforms to perform a worm-based analysis system. Unquestionably, such a kind of worm-on-a-chip platform, also known as ‘WoC’, can thus show us more potentialities from brand-new perspectives. WoCs have colonized a wide range of fields, encompassing drug screening [332,333,334], biosensors for diseases/environmental changes [335,336], neurosciences and disease modeling [337,338,339] and even parasitology [340,341,342].

## 12. Challenges and Future Insights

In parallel to the application value of OOC technologies in scientific investigation and the research realm, business in this field has boosted. Related leading companies have started to dominate the scene over the past 5 years [343], such as AlveoliX AG., Emulate, Inc., Hesperos Inc., MIMETAS Inc., Nortis, Inc., TissUse GmbH. In the same vein, the US Food and Drug Administration (FDA) announced in April 2017 that it had officially signed a multi-year cooperation agreement with Emulate Inc., expressing its high interest in using OOC cutting-edge technology to develop a testing platform for toxicological safety assessment [344]. These findings reveal the potential of applying OOC systems to human health assessments. In the next few years, this state-of-the-art technology will be able to integrate across diverse disciplines and parameters, encompassing stem cell technology, microenvironment and personalized medicine (for example, heart rate, breathing pattern, sub-stance abuse, etc.) in order to allow the construction of models of different genders, regions, ages, sexes, and diseases to minute minor physiological ailments and disorders, thereby promoting the development of precision health, also known as personalized health [345].

Regardless of all the advancements made with OOC technology in terms of individualized and personalized models, there remains the critical issue that the organ-level functional replication is still limited by the source of cells. In 2019, two pilot investigations led by Shiraishi et al. and Weiner et al. revealed that in the case of pulmonary alveolar model, the aspect of the long-term culture of primary human alveolar type I and type II epithelial cells is a particularly challenging hurdle [346,347]. For that reason, the organ-on-a-chip technology is faced with several blockages, such as limited availability and the inability to expand primary cells, requiring the establishment of cell cultures directly from healthy donors and/or patients and leading to an imminent boost of the cost of experiments and the difficulty of popularizing the technology. On the other hand, polydimethylsiloxane, ‘PDMS’, represents the crucial component of the OOC technology due to its high biocompatibility, oxygen permeability, and transparency. The PDMS chip devices can directly match conventional cell culture incubators and biological microscopes. However, a critical drawback of this silicone polymer is its high adsorbent potential of protein molecules on its surface [297,348], which may result in the supplement or stimulating endogenous and/or exogenous substance of the cell culture not fully interacting with the cells. In order to overcome this obstacle—the adsorption of non-specific proteins—some teams have tested and used other polymers, including polycarbonate (PC) [349], polystyrene (PS) [350], poly methyl methacrylate (PMMA) [299] and polylactic acid (PLA) [351]. More pros and cons of PDMS materials have been developed in detail by other investigations [348,352].

## 13. Concluding Remarks and Future Outlooks

Remarkable progress in terms of trends in food technology, life science, biomedical research, tissue bioengineering and biomechanical investigation in the past century has advanced our fundamental understanding of human physiology and the inter-organ crosstalk and intricate network, far beyond our imagination. Nevertheless, the ever-increasing knowledge in these active investigative areas has done remarkably little to improve our capability to mimic the complexity of human tissues, their vascularization, and the inter-organ crosstalk in experimental micro-architectural platforms.

Modeling changes of micro-engineered tissue function corresponds to another relevant hallmarks in the field of biomedical investigation. The appraisal of cellular function should go beyond concerns of live/dead viabilities or a change of protein expression. The ability to quantify the overall tissue in real-time fashion will boost the utility of OOC platforms. The standardization and optimization of OOC model systems to summarize human physiology and to obtain reproducible consistent outcomes are yet to be achieved. With ‘Good Cell Culture Practice’ (GCCP), the quality of cells in each unit of the OOC platform should be authenticated. The model should be compatible with the existing microscope and spectrophotometers for measurement. In this line, the integration of quite a few highly innovative electronic components seemed to be the most efficient tool in order to enable the analysis of biological molecules and the detection of cellular functional changes. This involves the implementation of transepithelial electrical resistance (TEER) sensors to non-invasively assess the endothelial/epithelial interface upon applied treatments on different cell lines and tissues. Accurately fastidious, highly miniaturized, and sophisticated, chip-integrated biosensors offer the potential to precisely quantify additional biological readouts, such as soluble molecules, and recreate specific procaryote-eucaryote microenvironment and all the associated outcomes.

Recently, the development of biosensors within OOC technology has witnessed a lightning progress. This category of biosensors can be functionalized and calibrated off-chip and then plugged into the chip for real-time in situ metabolite measurement. The above method of plug-in overcomes the major drawbacks of biosensors, such as short lifetime and reproducibility. For remote online monitoring of cellular parameters, revolutionary wireless portable sensor devices have been designed and developed, encompassing smart watches [353] and Google glasses [354]. They can be incorporated into OOC devices to control the parameters from any place and at any time.

The storage and transportation of chips represent critical parameters to preserve their functions for commercial use, and this is a challenge that needs attention. To succeed in the translation from laboratory to commercial platforms, alternative low-cost biomaterials for device fabrication are urgently needed for mass production at the industrial scale. Regarding the cell category, stem cell technology seems to be helpful for creating models based on an individual person. Likewise, creating a user-friendly platform will limit the call for expertise in cell seeding, device handling and manipulation in the future. Accordingly, leaders of pharmaceutical industries, academic institutions, regulatory agencies, and legislative structures must initiate a joint venture to launch and utilize the full potential of OOC technology.

We envision, in the near horizon, that future OOC platforms will have the ability to investigate the complex inter-organ-scale crosstalk to further expand knowledge on drug delivery, toxicity assessment, the study of sturdy infections, specific microenvironments reconstitution, and personalized medicine [355]. Indeed, there has been a relevant push towards developing body-on-a-chip devices and biosystems that entail multiple engineered tissues to analyze the potential indirect effects of metabolites, molecules, antibiotics and aliments. With the ability to connect multiple organs in a single miniaturized sophisticated micro-architecture model, it seems so promising to analyze complex ailments and disorders that encompass multiple organs and are influenced by the immune cells. We believe that nonstop progress in this field will direct future investigation into one single-organ and/or multi-organ function and provide a platform to yield precision medicine based on patient-derived cells or tissue biopsies and their microbiome depending on the tissue type.

To wrap up, it is urgent to call on all stakeholders of different disciplines, including experts in cell biology, 3D printing, microfluidics, microelectronics, immunohistochemistry, physiologically based pharmaco-kinetics modeling, artificial intelligence/machine learning, big data analysts and robotization, and to link them up for further accurate biomedical investigation. Additionally, connections to R&D laboratories in the medical, pharmaceutical, chemical, and food industries as well as legislative agencies will be the salient hallmark for ensuring that a system can be readily implemented and complies with regulatory, quality-related, and practical requirements.

## Figures and Tables

**Table 1:** The main discoveries/highlights in the organ-on-a-chip realm.

Year	Highlights
1991	The doorway to the ‘organ-on-a-chip’ opened with Dr. Kleber’s ‘patterned growth of neonatal rat heart cells in culture’
1997	The biophysical explanation of the cardiac conduction block, based as conducted on an in vitro cardiac model
1998	The emergence of polydimethylsiloxane (PDMS) as a template for microfluidic devices
2004	Functional maturation of rat cardiomyocytes induced by electrical stimulation
2006	Rise of the murine-induced pluripotent stem cells
2007	Establishment of ‘lung-on-a-chip’ device
2008	Establishment of ‘intestine-on-a-chip’ system
2010	The integration of a mechanically stretchable device for advanced lung-on-a-chip modeling
2012	3D-printed vascular network
2013	The inception of the ‘multi-organ-on-a-chip,’ by amalgamating 3D human liver and skin tissue co-cultures
2014	Polyurethane elastomer as a PDMS alternate template for microfluidic devices
2015	Development of kidney organoid
2016	Development of multi-organ-on-a-chip mirroring the in vivo microenvironment of lung cancer metastasis
2017	Integrating organoid technology and organ-on-a-chip engineering
2018	Projection-based 3D printing of cell patterning scaffolds with multi-scale channels to precisely mimic the invivo microchemical environment
2019	Understanding the foreign body response on a microfluidic platform on a chip

**Table 2:** OOC different platforms: Salient traits and chip performances (Adapted from [71], slightly modified).

OOC Platform	Key Aspects	Device recital/Applications	Reference
**Heart**	Human cardiomyocytes; Mechanical and electrical signals; Cardiomyocyte cluster; Heart dynamics.	Micro-engineered cardiac tissue; Can track the mechanical stimuli/responses and the electrical stimuli/responses of the heart; Helps measure in vitro contractile effects; Fabricating novel nanometric reinforced matrix to evaluate the cardiac contractility; The recent development of the Integrated heart/cancer-on-a-chip.	[72]
**Brain**	Central neuron system and the blood–brain barrier; Network activity; Signals from the various areas of the brain.	It paved the way for the research of neurodevelopmental studies, neurotoxicology, neuro-regeneration, and neuro-oncology studies; Determining the dynamic condition for the neuro- spheroids to develop; Multiregional brain-on-a-chip.	[73,74]
**Liver**	Liver sinusoid; Substance transport; Metabolism; Bile canaliculi; Hepatocyte–cell interaction; Hepatitis-B virus replication.	Designing co-cultures of hepatocytes and hepatic stellate cells to study cellular interaction without flow; Amalgamating the liver with modified sensors can help to predict mitochondrial dysfunction; Designing the three-dimensional cellular structure to develop hepatocyte, thus enabling us to assess the toxicity of drugs.	[75,76]
**Lung**	Intravascular thrombosis in lung alveolus; Alveolar-capillary interface; Respiratory dynamics.	Can visually identify and characterize the inflammation process during a bacterial response of our immune system; It helps to evaluate chronic lung diseases by tethering smooth muscle cells with epithelial cells; Aids in reproducing clinical tests more accurately and precisely.	[77,78]
**Kidney**	Tissue engineering of a bioartificial renal tubule; Albumin resorption; Functional coupling of jejunum, liver, kidney (PT), skeletal muscle, and neurovascular model; Hemodialysis; Functional coupling of renal tubular and vasa recta function.	Analyzing drug-induced nephrotoxicity screening for early detection of drug-induced kidney injury; Artificial proximal kidney probe used for nephrotoxicity probes; To predict the absorption along with the metabolism of drugs, for faster clinical pharmacodynamics.	[32,79]
**Skin**	Absorption; Barrier function; Air-to-liquid interface.	Complements the provision of more precise treatment for inflammation and edema; It helps to analyze the effect of cosmetics and chemicals that are applied to the skin; It can bypass the problems of inconsistent seeding, epithelial damage, and dermal matrix contraction.	[80,81]
**Gut**	Epithelial barrier mimicry; Microbial interface; peristalsis motion; Gut viral infection.	Understanding the pathophysiology when a foreign body enters the human cell; To provide an alternative to in vivo testing by modeling gamma radiation injury; The complex system enables the technology to provide an artificial environment to test polarized infection.	[82,83]

**Table 3:** Summary of most relevant OOC platforms, their applications and the associated disease models.

OOC Platform	Cell Types	Disease Models	Reference
**Brain and Blood–Brain Barrier (BBB)**	Endo, pericyte, astrocyte	Inflammatory response due to the presence of endotoxins	[201,202]
	Neuron, microglial, astrocyte, pericyte, endo (all iPS)	Parkinson disease	[203]
	Endo, astrocyte, neuron (all iPS)	Huntington disease, MCT8 deficiency	[204]
	Endo (iPS), pericyte, astrocyte	BBB transport (to clinically mimic drug and antibody transport)	[201]
	Endo, pericyte, neuro stem, fungal	Fungal meningitis (to clinically impersonate fungal invasion of BBB)	[205]
**Cardiac tissue**	Cardiomyocyte (iPS)	Cardiac fibrosis, drug-/stress-induced hypertrophy, arrythmia	[206,207]
	Cardiomyocyte (iPS)	Cardiotoxicity	[208]
	Cardiomyocyte (iPS), endo (3D-printed)	Heart contractility, cardiotoxicity	[87]
**Artery and blood vessels**	HS-27a, HUVECs, Stromal cells	The dose dependent vasoconstriction	[178,209]
	Aortic smooth muscle (iPS)	Progeria, inflammation, mechano-sensitivity	[210]
	Endo	Thrombosis	[211]
	Epi, endo, cancer	Inflammation	[212]
	Endo, kidney cancer (patient)	Tumor angiogenesis	[213]
	Aortic smooth muscle	Aortic valve insufficiency, mechano-sensitivity	[214]
	Aortic smooth muscle, endo, immune	Atherosclerosis, vascular stenosis (to clinically emulate vascular inflammation)	[215]
	Stromal cell, bone marrow mononuclear	Implant-associated metal accumulation in bone (a clinical mimicry of implant toxicity)	[216]
**Lymphatic vessels**	Lymphatic endo	The dose dependent inhibition of lymphatic growth.	[217]
	Lymphatic endo, breast cancer	Breast cancer (to clinically reproduce Breast cancer lymphangiogenesis)	[215]
**Microvasculature**	HS-27a, HUVECs, Stromal cells	Increased gene expression, inhibition of angiogenesis, reduced vascular sprouting	[209]
**Eye and Blood retinal barrier**	Retinal pigmented epithelial, endo	Inhibition of angiogenic sprouting by bevacizumab	[218]
	Retinal pigmented epithelial, 7 retinal (iPS org)	Retinopathy	[219]
	Retinal pigmented epithelial, 7 retinal (iPS org)	Gene therapy delivery (a clinical mimicry of AAV vector delivery)	[220]
**Lung and Airway passages**	Epi, endo	The epithelial stimulation with inflammatory cytokine tissue necrosis factor alpha	[221,222]
	Epi (line)	The toxicity due to the silica nanoparticles in the pulmonary region	[198,223]
	Epi, endo	Pulmonary oedema caused via the interleukin-2 which is detected by the leakage of fluids	[120,224]
	Epi, endo	The infiltration from the neutrophils	[225,226]
	Epi (line), endo, immune	Virus infection (SARS-CoV-2), inflammation	[227]
	Epi, endo, cancer	Lung cancer	[212]
	Epi	COPD induced by smoke	[228]
	Epi (line), endo, immune	Asthma, COPD	[191,229]
	Epi	Mechanical injury to airway cells	[230]
	Epi (line), endo, immune, bacteria	Cystic fibrosis, inflammation, bacterial infection	[231]
	Epi, endo, immune	Virus infection (influenza, pseudotyped SARS-CoV-2), inflammation	[232]
**Skin**	Keratinocyte	Model for wound healing, inflammation, repair, irritation, ageing and shear stress studies	[233]
**Gut**	Epi (org), endo	Intestinal differentiation	[145]
	Epi (line), endo	The epithelium infected by virus coxsackie B1 The pathogen-induced injury	[234]
	Epi (line), endo	The peristaltic mechanical deformation-induced bacterial out-growth	[235,236]
	Epi (line), bacteria	Host-microbiome interactions (to clinically simulate effects of microbiome metabolites on host)	[237]
	Epi (line)	The loss of the barrier function caused by staurosporine and aspirin	[238]
	Epi (line), endo, lymphatic, endo, immune, bacteria	Bacterial infection and inflammation	[144]
	Epi (line)	Enteric virus infection (to clinically mimic infection-associated injury)	[239]
**Gut**	Epi (org), immune	Inflammatory bowel disease (IBD)	[240]
	Epi (org), endo, immune, virus	Enteric virus infection	[241]
	Epi (line), endo, virus	SARS-CoV-2 virus infection	[242]
	Epi (org), bacteria	Bacterial infection, mechano-sensitivity (to clinically mimic *Shigella* infection)	[243]
	Epi (line), endo,	Radiation injury	[244]
**Kidney**	Epi	The injury in the cells, Renal transport and nephrotoxicity	[79,245]
	Epi, endo	Renal transport, hyperglycaemia (to clinically impersonate renal reabsorption and drug efficacy)	[246]
	Endo (line), podocyte (line)	The glomerular injury induced by Adriamycin The elevated perfusion rate caused by hypertensive nephropathy	[247]
	Epi (line)	Nephrotoxicity	[248]
	Endo, podocyte (iPS)	Filtration barrier (to clinically mimic urinary clearance and drug toxicity)	[129]
**Liver**	Hep	The drug toxicity quantification (Benzbromarone) and other model drugs	[249,250]
	Hep	The inhibition of the glucogenesis and hepatic clearance	[190]
	Hep	Drug metabolism	[251]
	Hep	Inflammation effects on drug metabolism	[252]
	Hep, Küpffer	CYP450 metabolism, drug–drug interactions	[253]
	Hep	Drug- and toxin-induced liver injury	[254]
	Hep, endo, hepatic stellate, Küpffer	Drug-induced liver injury	[255]
	Hep, Küpffer	Virus (Hepatitis B) infection, inflammation (a clinical reproduction of viral infection and associated injury)	[256]
**Pancreas**	Whole isolated pancreatic islets	Diabetes mellitus (to clinically mimic glucose-sensitive insulin secretion)	[257]
**Tumors**	Epi, endo	Investigation of chemical chemotherapeutic process	[258,259]
	Epi, endo	Deciphering the survival and proliferation of malignant cells	[259]
	Epi, endo	Breast cancer (a clinical mimicry of mutation-induced cancer progression and angiogenesis)	[260]
**Placenta**	Trophoblast (line), endo	Placental barrier	[261]
**Uterus**	Epi, stromal	Endometrial remodeling (a clinical mimicry of uterine contraception and drug efficacy)	[262]
	Epi, endo, stromal	Endometrial remodeling (to clinically reproduce menstrual cycle-dependent and endometrial differentiation	[263]
**Teeth**	Dental stem, dentin	Dental material toxicities (a clinical simulation of biomaterials toxicity)	[264]
	Dental stem, dentin, bacteria	Biofilm formation	[265]

AAV: adeno-associated virus; COPD: chronic obstructive pulmonary disease; CoV: coronavirus; endo: vascular endothelial cell; epi: epithelial cell; hep: hepatocyte.

**Table 4:** Multi-organ-on-a-chip: Key aspects and chip presentation.

OOC Platform	Key Aspects	Chip Recital	Reference
**Multi-Organ-On- Chip**	The microphysiological systems for drug development studies; Organ–organ interaction; The multi-functioning of the organ system (for instance, metabolism of drugs in the small intestine together with the metabolism by the liver and excretion by the kidney).	Reducing drug throughput screening; Enhancing high content screening when loaded with a small amount of drugs, thereby tuning it economically viable; It can control the microenvironment, thereby accurately predicting the efficacy of the drug. However, the emerging trends of multi-organ-on-a-chip face several drawbacks as compared with the ‘single-on-a-chip.’ While some of them are related to the organ scaling and vascularization of tissues, others are associated with immune components and accurately considering the cell cycles (e.g., circadian cycle).	[286,287]

**Table 5:** Pros and cons of OOC technology.

Technology	Applications in Life Science	Size	Materials of Fabrication	Biological Samples
**OOC**	Study human physiology; Disease modeling; Drug screening; Drug development and delivery; Toxicity tests and assessment; Personalized medicine.	Few square centimeters	Biocompatible and cyto-compatible materials such as polymers (e.g., PDMS); Glass; Biological materials (e.g., proteins, cells etc.).	Cells, Spheroids, Organoids, Tissue biopsies
**OOC**	**Pros**		**Cons**	
	High ability to be integrated with miniaturized sensors and actuators; Advantages linked with new microfabrication techniques (e.g., soft lithography, 3D bioprinting and etc.); Such micro-architecture needs small volume of sample and reagents consumption; Real-time and on chip analysis; Recreating of specific microenvironments; Precise control over microenvironment; Study of prokaryote–eukaryote interactions.		Need well-trained experts to collect and interpret data; Non-defined protocols; Current problems with fabrication materials (e.g., absorption of small molecules by PDMS) Current platforms mostly are not automated; Problem linked with cell clogging and bubbles formation in microchannels; Cell damages due to shear stress.	

**Table 6:** Examples of biosensing systems in the monitoring of relevant biomarkers in OOC models.

Nature of Biosensor	OOC Platform	Biomarker(s)	Detection Threshold	References
Optical	Pancreas	Insulin	µg/mL	[307]
Electrochemical	Heart	Creatine kinase	pg/mL	[308]
Electrochemical	Liver/Heart	Creatine kinase, albumin, and GST-α	ng/mL	[309]
Electrochemical	Muscle	IL-6 and TNF-α	ng/mL	[310]

IL: interleukin; TNF-α: Tumor Necrosis Factor-α; GST-α: Glutathione *S*-Transferase-α.

**Table 7:** Examples of hydrogel-based OOC platforms.

Hydrogel-Based OOC	Hydrogel Types	References
Hydrogel chips	GelMA	[311]
	PEGDA	[312]
	Fibrin/gelatin	[173]
	GelMA/alginate	[88]
Vascularized networks	Collagen	[154]
	PoMaC	[63]
	Fibrin/gelatin	[313]
	Alginate/GelMA/gelatin	[314]
Tissue–tissue interfaces	Collagen I	[315]
	Matrigel	[316]
	Chitosan	[317]
	Collagen I/matrigel	[318]
	Gelatin/agarose	[319]
Parenchymal tissues	Alginate	[320]
	Agarose/alginate/HA/PEG	[321]
	GelMA/DLM	[117]
	Collagen I/matrigel	[322]

**Table 8:** List of the most important start-up leaders in the OOC field and highlights of each technology (Adapted from Azizipour et al. [328] with slight modifications).

Start Up’s Name	Field of Investigation	Applications	Cell Source	Salient Pros of the Technology	Year
**CN BioInnovations**	OOC, Liver-on- a-chip, Body- on-a-chip	Human physiology modeling, Liver diseases modeling, Preclinical drug discovery, Toxicity assessment, Drug metabolism	Primary human cells, Tissue or Organ Slices, iPSCs, Immortalized cell lines	Multi organ studies, Portable and compact device, Programmable flow rate, Open well plates	2009
**TissUse**	OOC, Body-on- a-chip	Disease modeling, Personalized medicine, Toxicity experimental trials, Drug development, Application in pharmaceutical and cosmetic research	Cell lines, Human primary cells, Biopsies	Multi-organ platforms, Rapid prototyping, Compatible with tissue imaging, Application of physiological sheer stress, Long- term performance	2010
**Nortis**	OOC, Kidney, Brain, Heart, Liver, Immune system and blood vessels- on-a-chip	Disease modeling, Cancer investigations, Drug delivery testing, Study Alzheimer’s disease and ageing, Toxicity tests	Human derived tissue models	Perfusion system, Standard cell culture incubator,	2012
**MIMETAS**	OOC	Disease modeling, Drug testing, Toxicity tests, Personalized medicine	Human cells, patient derived cells or tissues	Organo-Plates (a microfluidic 3D cell culture plate), 3D co-culture, Biomimetic, compatible, Easy to use	2013
**AxoSim**	OOC, Nerve-on- a-chip	Preclinical testing, 3D cell culture, Neurotoxicity tests, Neurodegenerative diseases	Primary cultures, Organoids	Biomimetic human tissues, Combination of neurons, astrocytes, and oligodendrocytes.	2014
**Emulate, Inc.**	OOC, Lung, Bone marrow, Kidney, Brain, Blood vessels and intestine- on-a-chip	Personalized medicine, Disease modeling, Drug screening, Study human physiological responses	-	OOC devices personalized with individual patients’ stem cells, Stretchable biochip, Flexible and dynamic environment by continuous fluid flow and mechanical stretch	2014
**SynVivo, Inc**	OOC, Blood-brain-barrier- on-a-chip	Drug discovery, Toxicity assessment, Targeted drug delivery, Cancer research	Human cells	Emulate dynamic microvascular environment, Real-time visualization, Controlled condition, 3D co-culture model	2014
**TARA Biosystems**	OOC, heart-on-a-chip	Cardiac Toxicology, Precision Cardiology, Heart Failure Drug Discovery, Drug development, Study healthy and disease models	iPSCs derived cardiomyocytes	Cardiac tissue models, Patient derived disease models	2014
**Hesperos**	OOC, Multi-organ-on-a-chip (heart, liver, lung, brain, skin, muscle, kidney, pancreas, bone marrow)	In vitro trials, Drug discovery, Toxicity tests, PK/PD modeling	Human stem cells	Pumpless platform, Restructure muscle and tissue function, neural and inter-organ communication, Personalized human-on-a-chip platform, Possibility to add immune cells in multi-organ-platform	2015
**AlveoliX**	OOC, Lung-on-a-chip	Drug discovery, Disease modeling, Personalized medicine	Human cell lines	In vitro models inspired by nature, Reproduce lung breathing motion and stretching, Elastic and ultrathin membrane	2015
**BEOnChip**	OOC	Disease modeling, In vitro tests, Drug screening	Diverse human cell lines	Long-term 2D or 3D culture under flow condition, 2D-3D co- culture, Emulation of physiological environments involving flow and shear stress	2016
**Biomimx**	OOC, Heart-on-a-chip; Cartilage-on-a-chip	Drug screening, Drug cardiotoxicity assessment, Anti-cardiac dysrhythmia medications efficiency, Discovery of anti-osteoarthritic drugs	Cardiomyocytes derived from human iPSCs, Human cells	3D co-culture, Mechanical stimulations, Human cardiac tissue, Human osteo-arthritic cartilage, Tailored OOC	2017
**BI/OND**	OOC, BI/OND’s microfluidic plate	In vitro tests, Drug discovery, Drug delivery	Human cells, Organoids, Patient derived cells or tissues	Dynamic cell culture environment by providing mechanical stimulation and continuous fluid flow, two compartments connected by a porous membrane BI/OND’s plate to run up to six cultures in parallel, 3D and 2D models	2017

iPSCs: Induced Pluripotent Stem Cells; OOC: Organ-On-Chip; PK/PD: pharmacokinetic/pharmacodynamic.
